# Supplementary material for: Metformin Protects against Radiation-Induced Acute Effects by Limiting Senescence of Bronchial-Epithelial Cells
Source: Int J Mol Sci. 2021 Jun 30;22(13):7064. doi: 10.3390/ijms22137064 (PMC8268757; doi:10.3390/ijms22137064)
Supplement: Supplementary file 1 [file ijms-22-07064-s001.zip › ijms-1273852-supplementary.pdf]

Supplementary Information

Metformin Protects Against Radiation-Induced Acute Effects by Limiting Senescence of Bronchial-Epithelial Cells

Christine Hansel [1], Samantha Barr [1], Alina V Schemann [1], Kirsten Lauber [2,3,4], Julia Hess [4,5], Kristian Unger [4,5], Horst Zitzelsberger [4,5], Verena Jendrossek [1] and Diana Klein [1,*]

1 Institute of Cell Biology (Cancer Research), University Hospital, Essen, University of Duisburg-Essen, Essen, Germany; Christine.Hansel@uk-essen.de (C.H.); sjbarr@wisc.edu (S.B.) Alina.Schemann@gmail.com (A.S.); Verena.Jendrossek@uk-essen.de (V.J.)

2 Department of Radiation Oncology, University Hospital, LMU München, Munich, Germany; Kirsten.Lauber@med.uni-muenchen.de (K.L.)

3 German Cancer Consortium (DKTK), Partner site Munich, Germany

4 Clinical Cooperation Group 'Personalized Radiotherapy in Head and Neck Cancer' Helmholtz Zentrum München, German Research Center for Environmental Health GmbH, Neuherberg, Germany; julia.hess@helmholtz-muenchen.de (J.H.); unger@helmholtz-muenchen.de (K.U.); zitzelsberger@helmholtz-muenchen.de (H.Z.)

5 Research Unit Radiation Cytogenetics, Helmholtz Zentrum München, German Research Center for Environmental Health GmbH, Neuherberg, Germany

* Correspondence: Diana.Klein@uk-essen.de (D.K.); Tel.: +49(0)20172383342

Supplementary Figures

Supplementary Figure S1

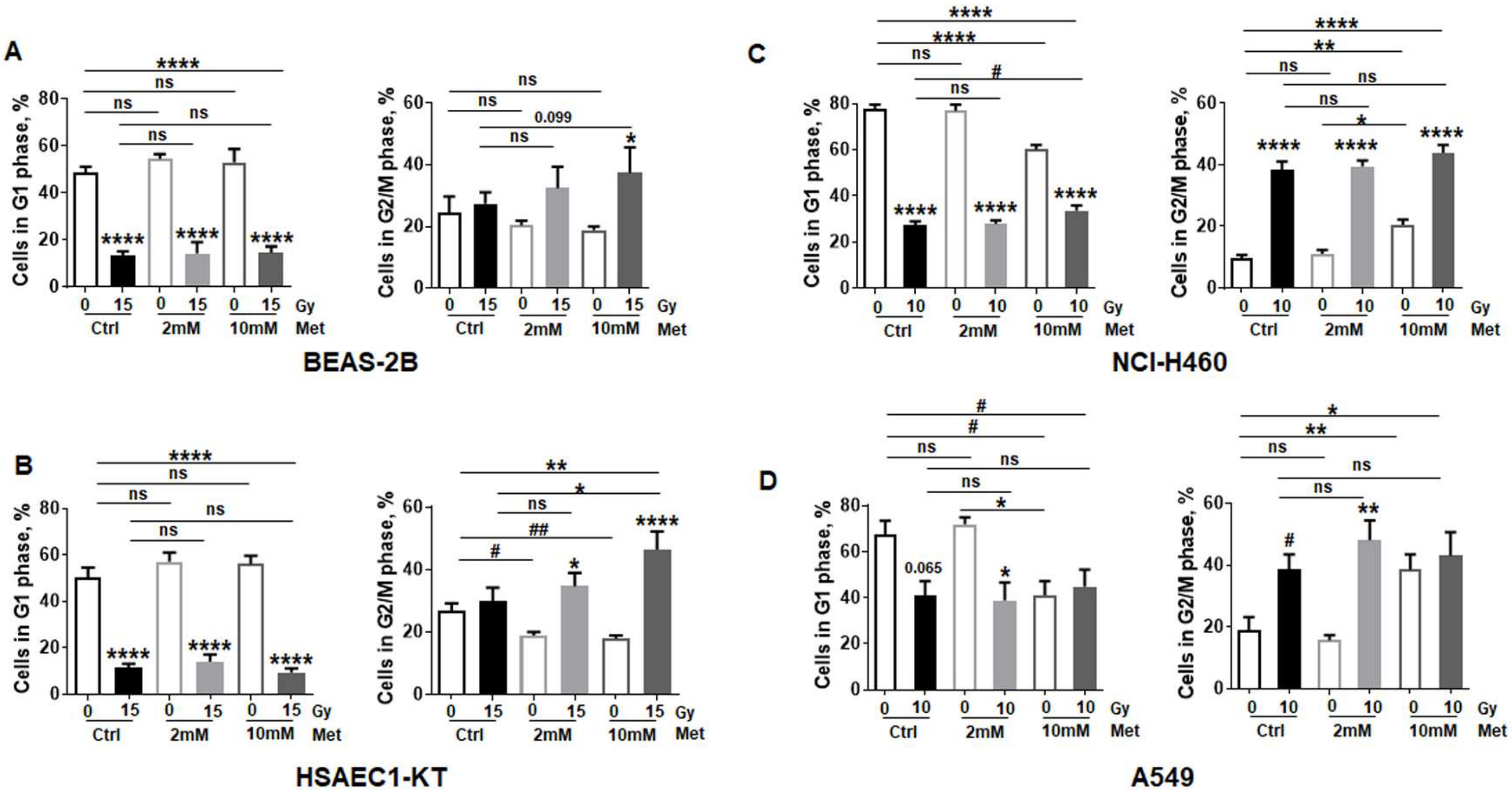

Supplementary Figure S1. Cell cycle analyses. Lung epithelial cell lines derived from normal lung epithelium (BEAS-2B: human bronchial epithelial cells, **A**; HSAEC1-KT: human small airway epithelial cells, **B**), and non-small cell lung cancer cells (NSCLC; NCI-H460: human epithelial large cell lung carcinoma; **C** and A549: human alveolar basal epithelial adenocarcinoma; **D**) were cultured in normal growth media supplemented with metformin (2 and 10 mM) or vehicle control (Ctrl) 2 hours prior radiation treatment with 0Gy, 15Gy (**A, B**) or 10Gy (**C, D**). G_1 and G_2/M cell cycle phases (96 hours post treatment), as presented in Figure 4A, D (non-malignant lung epithelial cells) and Figure 8C, G (NSCLC cells), were depicted in separate graphs. Graphs consist of data from 3-7 individual experiments (with SEM). P-values indicate: * $p \leq 0.05$, ** $p \leq 0.01$, **** $p \leq 0.0001$ by two-way ANOVA with Tukey's multiple comparison test, and additionally by unpaired (two-tailed) t-tests depicted as # p≤0.05 and ## p≤0.01.

Supplementary Figure S2

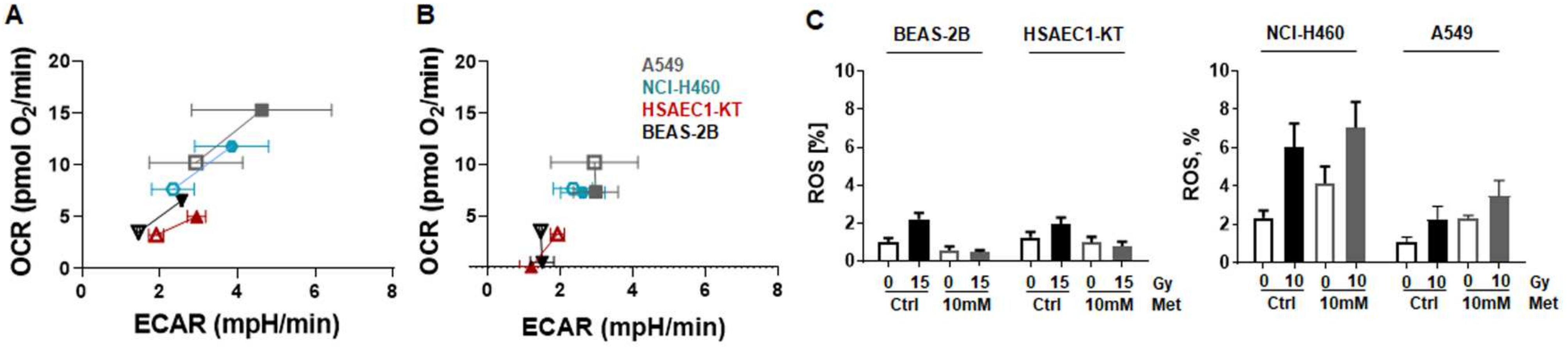

Supplementary Figure S2. Metformin differentially impacts on RT-induced cellular stress in normal and malignant lung epithelial cells as determined by cell metabolisms, specifically mitochondrial respirations and glycolytic activities together with cellular ROS levels. The relative utilization of the two energy pathways mitochondrial respiration and glycolysis, as estimated by oxygen consumption rates (OCR) and extracellular acidification rates (ECAR) over time, in normal (BEAS-2B and HSAEC1-KT) and malignant (NCI-H460 and A549) lung epithelial cells were summarized upon both baseline (baseline phenotype) and stressed (stressed phenotype) conditions. (**A**) Basal levels (open symbols) of untreated cells and stressed levels (closed symbols) after the addition of oligomycin, FCCP, rotenone and antimycin A are shown. Whereas in normal epithelial cells primarily mitochondrial oxidative phosphorylation is efficient to generate energy for homeostasis, the increasing energy demands of malignant epithelial cells are accomplished by increased mitochondrial respiration as well as glycolysis as determined by increased mitochondrial respiration and glycolysis rates. The response to induced energy demands, known as the cell's metabolic potential, is higher in malignant epithelial cells compared to normal epithelial cells. (**B**) Basal levels (open symbols) of untreated cells and stressed levels (closed symbols) after 24 hours of combined radiation treatment with metformin are shown. The combined treatment reduced the metabolic potential of both malignant epithelial cell types, which mainly rely on oxidative phosphorylation and less on glycolysis. Normal epithelial lung cells turned out to be more resistant following metformin treatment (in combination with RT), most likely due to the lower steady state levels. Accordingly, increased ROS levels following metformin treatment (in combination with RT) were estimated in malignant epithelial cells compared to normal epithelial cells (**C**). ROS in turn critically regulates (apoptotic) cell fate decisions. Malignant epithelial cells, that highly proliferate and need more energy, showed increased cellular stress levels, while the metabolic potential decreased upon combined metformin and radiation treatment, and thus, preferentially undergo cell death.

Supplementary Figure S3

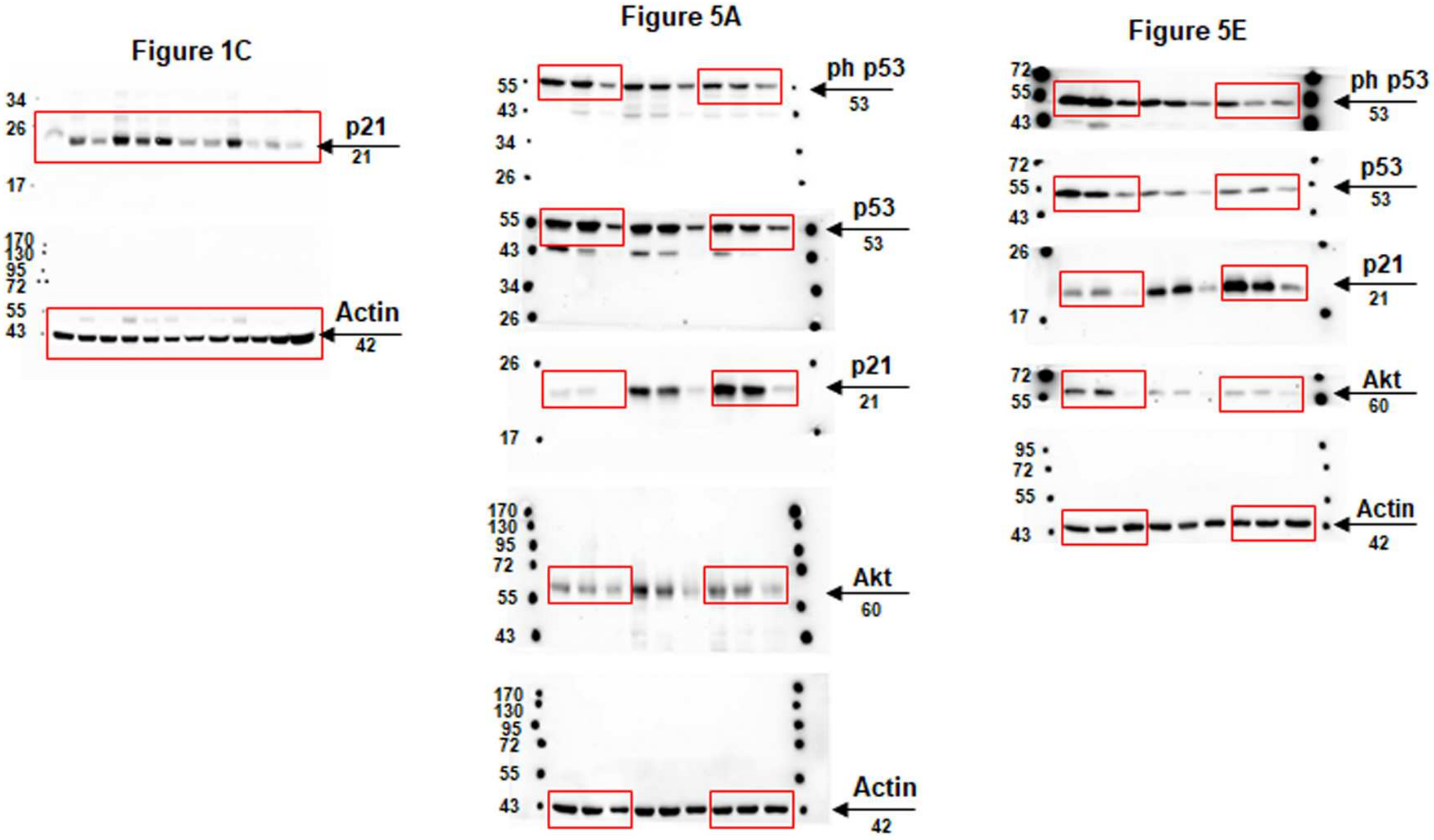

Supplementary Figure S3. Full gels of cropped gels (emphasized by a red rectangle) as shown in Figure 1C, 5A, 5E. Equal protein amounts were loaded. Beta-actin was included as a loading control.
